# Association between lymphocyte subsets and cytomegalovirus infection status among patients with systemic lupus erythematosus: A pilot study

**DOI:** 10.1097/MD.0000000000016997

**Published:** 2019-09-27

**Authors:** Ling Qin, Zhifeng Qiu, Evelyn Hsieh, Taoran Geng, Jiuliang Zhao, Xiaofeng Zeng, Lu Wan, Jing Xie, Rayoun Ramendra, Jean Pierre Routy, Taisheng Li

**Affiliations:** a Department of Infectious Diseases, Peking Union Medical College Hospital; b Center for AIDS Research; c Clinical Immunology Center, Chinese Academy of Medical Sciences & Peking Union Medical College, Beijing, China; d Section of Rheumatology, Allergy & Immunology, Department of Internal Medicine, Yale School of Medicine, New Haven, CT; e Department of Rheumatology; f Department of Internal Medicine, Peking Union Medical College Hospital, Chinese Academy of Medical Sciences & Peking Union Medical College, Beijing, China; g Department of Microbiology and Immunology, McGill University; h Chronic Viral Illnesses Service and Division of Hematology, McGill University Health Centre, Montreal, QC, Canada.

**Keywords:** CD4+ T cells, CMV infection, systemic lupus erythematosus

## Abstract

This study aimed to determine the association between different lymphocyte subsets and cytomegalovirus (CMV) infection status in patients with systemic lupus erythematosus (SLE). We performed a retrospective study among SLE patients with CMV infection and collected patient socio-demographic and clinical characteristics, as well as their recorded circulating lymphocyte subsets. Univariate and multivariable logistic regression analyses examined the relationship between CMV infection status and lymphocyte subset counts. We included 125 hospitalized patients with SLE, consisting of 88 with documented CMV infection and 37 without any evidence of CMV or other infections. Among the 88 CMV-infected patients, 65 (73.8%) patients developed CMV disease and 23 (26.2%) presented as CMV viremia. Compared to uninfected patients (1520 ± 101 cells/μL), lymphocytes remained stable among those with CMV viremia (1305 ± 272 cells/μL, *P* = .995). However, compared to their uninfected counterparts, there was a marked decrease in lymphocytes among patients with CMV disease (680 ± 513 cells/μL, *P* < .001). Analysis of lymphocyte subsets via flow cytometry showed that CD4+ T cell, CD8+ T cell, and natural killer cell counts were lower among those with CMV disease compared to those with CMV viremia and those without infection. Further, multivariable analysis showed that total lymphocyte (odds ratio [OR] 0.999, 95% confidence interval [CI] 0.998–1.000, *P* = .007) and CD4+ T cell counts (OR 0.99, 95% CI 0.992–0.998, *P* = .003) were negatively associated with CMV disease. Our findings support a potential inverse relationship between lymphopenia, specifically CD4+ T-cell lymphopenia, and CMV disease among hospitalized SLE patients.

## 1 Introduction

Severe infection represents one of the main causes of hospitalization among patients with systemic lupus erythematosus (SLE), and significantly contributes to morbidity and mortality in this population.^[1–3]^ Data from France and Canada indicate that infections are the third most common cause of death among patients with SLE^[5,6]^ and in China, infections are the leading cause of death in this group.^[7]^ The few studies that have explored the spectrum of opportunistic infections (OIs) in patients with SLE suggest cytomegalovirus (CMV) is an important pathogen in this population. We previously found that CMV is the most common OI (61.1%) encountered among hospitalized patients with SLE in Beijing, followed by fungal infections, including *Pneumocystis jirovecii* pneumonia (29.6%). In addition to patients with SLE, CMV infection is also recognized as one of the most common complications among immunocompromised individuals in general.^[4,5]^ Furthermore, a potential role for CMV in the pathogenesis of SLE has been suggested by some studies.^[6,7]^ Finally, although the presence of CMV infection can be detected via CMV antibodies, antigens and/or pathological findings, it remains difficult to differentiate between relapse of SLE and clinically relevant CMV infection against the backdrop of autoimmune abnormalities.^[8]^

The clinical manifestations of CMV infection among SLE patients can vary from no symptoms to serious organ damage,^[9]^ and can present in patients with newly diagnosed SLE or those on stable chronic low-dose immunosuppressive therapy.^[8]^ One of the possible mechanisms responsible for the wide spectrum of clinical presentations in CMV-infected patients with SLE is the alteration of T cells.^[10]^ It is well-known that T-cell lymphopenia is observed in SLE patients,^[11–13]^ and that lymphopenia secondary to disease activity may also contribute to susceptibility to infection.^[12]^ Quantitative and/or qualitative defects of T-regulatory cells, as well as cytokine imbalance (eg, decreased IL2 and increased IL17 production by CD4+ T cells) contribute to increased inflammation and impaired immunity against infections.^[14]^ Furthermore, circulating anti-lymphocyte autoantibodies may lead to decreased numbers of natural killer (NK) cells,^[15]^ and B cell and immunoglobulin disorder^[16]^ have been described, contributing to the decreased ability of SLE patients to fight infections.

Our prior study found that lymphocyte subsets, including NK cells, T cells, and B cells, were decreased among SLE patients with OIs compared with uninfected patients with SLE.^[17]^ This finding was consistent with other studies that directly measured specific lymphocyte subsets, such as CD4+ T lymphocytes,^[18,19]^ and found CD4+T lymphocyte levels decreased after patients developed severe OIs. However, to our knowledge, no studies have specifically examined the relationship between lymphocyte subset counts and different clinical presentations of CMV infection. Given the potential utility of lymphocyte subsets as clinical biomarkers to distinguish between different clinical states of CMV infection, we leveraged a pre-existing Chinese lupus registry and designed the present pilot study to estimate counts of circulating lymphocyte subsets among SLE patients with CMV viremia and CMV disease as compared with those without infection and hypothesized that levels of lymphocyte subsets would be lowest among those with active CMV disease.

## 2 Methods

### 2.1 Study design

We performed a retrospective case-control study of patients with SLE from the Chinese SLE treatment and research group (CSTAR)^[20]^ who were hospitalized at Peking Union Medical College Hospital, a large, tertiary care hospital in Beijing, China from December 2013 through December 2016.

### 2.2 Study population

CSTAR is a nation-wide multicenter Chinese registry of patients with SLE. The data are collected online from 104 rheumatology centers, spanning 30 provinces in China. Adult patients (individuals >18 years of age) are eligible for inclusion in CSTAR if they have an established SLE diagnosis, according to the systemic lupus international collaborating clinics’ classification criteria.^[21]^ Details of CSTAR have previously been described.^[20]^ For the purposes of this analysis, patients were eligible for inclusion if they were enrolled in CSTAR and had peripheral blood lymphocyte subset screening at the time of admission. Exclusion criteria consisted of prior use of cell-based or biologic agents for other autoimmune conditions in the past (eg, rituximab or tocilizumab for rheumatoid arthritis).

CMV infections in this study were diagnosed according to the Guidelines for Prevention and Treatment of Opportunistic Infections in HIV-Infected Adults and Adolescents.^[22]^ Patients were classified in the CMV Viremia group if they had no CMV-related symptoms but had detectable CMV DNA levels in the blood (≥500 copies/mL) using quantitative real-time polymerase chain reaction (PCR) detection. Patients were classified in the CMV Disease group if CMV viremia was accompanied by clinical signs (eg, fever, pneumonia, or retinitis) of CMV with and without tissue biopsies. Patients in the Uninfected Control group were those without detectable CMV DNA in blood and no symptoms or signs suggesting other infections at and during the hospitalization.

### 2.3 Clinical data

Data regarding sociodemographic characteristics, SLE diagnosis history, erythrocyte sedimentation rate (ESR), C reactive protein (CRP), complement 3 (C3), complement 4 (C4), systemic lupus erythematosus disease activity index (SLEDAI) score, and usage and dose of corticosteroids and/or immunosuppressive drugs were extracted from each patient's medical chart. The peripheral total lymphocytes and lymphocyte subset counts of sex- and age-matched healthy individuals (n = 60), randomly selected from a previously published cross-sectional study of 1068 Chinese healthy individuals, was included to provide reference values for each parameter.^[23]^

### 2.4 CMV detection

DNA extraction of the plasma samples (100 μL) was performed using the CMV DNA Diagnostic Blood kit, a quantitative real-time PCR detection of CMV DNA was performed on an Roche LightCycler 480 Detection System using Therma-Base Taqman technologies.^[24]^ CMV DNA viral loads were expressed in copies/mL with a threshold level of 500 copies/mL.

### 2.5 Flow cytometry

Immunophenotyping of blood lymphocyte subsets was analyzed by flow cytometry (Epics XL flow cytometry; Bechman Coulter) as previously described.^[25]^ In brief, freshly collected EDTA anti-coagulated whole blood was incubated and tested with a panel of monoclonal antibodies labeled with fluorescein isothiocyanate/phycoerythrin/peridinin chlorophyll protein and directed against combinations of CD3/CD8/CD4, CD3/CD16CD56/CD19 and isotype controls (Immunotech, France). Cell counts of lymphocyte subsets were calculated using a dual-platform method with the white blood cell counts and lymphocyte differentials obtained from routine complete blood count testing of the same specimen collected on the same day.

### 2.6 Statistical analysis

All analyses were performed using SPSS software (SPSS for Windows version 16.0, SPSS Inc, Chicago, IL). The Kolmogorov–Smirnov test was used to examine the cumulative distribution functions of the samples. Reference ranges were calculated using mean ± 2 standard deviations for parametric data or median for non-parametric data. For normally distributed continuous variables, the Student *t* test and analysis of variance were used for comparisons between 2 or 3 groups, respectively. The Wilcoxon rank-sum test was used for comparison of nonparametric variables. Chi-square or Fisher exact tests were applied as appropriate for comparison of categorical variables. We further performed 2 separate logistic regression analyses using CMV viremia and CMV disease as dependent variables, respectively. For each dependent variable, we first fit separate univariate models to examine the relationship between the dependent variable and individual lymphocyte subsets. We then further adjusted each model for the following baseline characteristics to generate a multivariable model: age, sex, ESR, CRP, C3, C4, SLEDAI, duration of SLE, corticosteroid use, use of cyclophosphamide (CPM), CMV viral loads and each lymphocyte subset (including NK cell, B cell, CD4+ T, and CD8+ T lymphocyte) and occurrence of CMV infection, including CMV viremia and CMV disease. We fit the multivariable models using backward elimination beginning with all variables that were hypothesized to be related to CMV infection. All tests performed were 2-tailed, with *P* < .05 considered to be statistically significant.

## 3 Results

### 3.1 Demographic characteristics of study participants

A total of 125 patients with SLE were included in the study, comprised of 23 patients with CMV viremia, 65 patients with CMV disease, and 37 patients without infections. In the overall study population, the average age of participants was 32.7 ± 11.8 years, 88.8% were women, and average duration of SLE was 60.2 ± 55.6 months. Demographic and clinical characteristics of the study participants by group are shown in Table 1. Of the 65 patients with CMV disease, the most common manifestations were persistent fever (48), pneumonia (24), pancytopenia (10), elevated liver enzymes (5), retinitis (1), and colitis (1). Patients were most commonly treated with CPM (n = 54), followed by hydroxychloroquine (HCQ, n = 37), mycophenolate mofetil (MMF, n = 24), cyclosporine (CSA, n = 10), and azathioprine (AZA, n = 4). Given the small number of patients treated with AZA, it was excluded from treatment-related analyses.

**Table 1:**
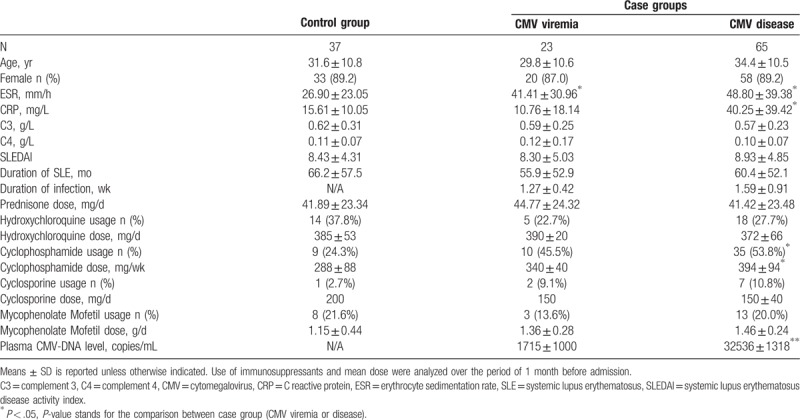
Demographic characteristics of patients with SLE in this study.

As shown in Table 1, compared to patients in the control group, a higher ESR level was observed among patients with CMV viremia (41.41 ± 30.96 mm/h vs 26.90 ± 23.05 mm/h, *P* = .037). No significant differences were observed between patients in the control group and those with CMV viremia, in terms of mean age, the proportion of females, complement levels, CRP levels, duration of disease, SLEDAI score, dose of prednisone, and immunosuppressive drug use. By contrast, compared with uninfected controls, patients with CMV disease showed a higher ESR (48.80 ± 39.38 mm/h vs 26.90 ± 23.05 mm/h, *P* = .006), CRP (40.25 ± 39.42 mg/L vs 15.61 ± 10.05 mg/L, *P* = 0.002), were more likely to use CPM (53.8% vs 24.3%, *P* = .006) and at higher doses (394 ± 94 mg/wk vs 288 ± 88 mg/wk, *P* = .002). However, no significant differences were observed between these 2 groups with respect to age, sex, complement levels, SLEDAI score, duration of SLE, and usage of steroid dose before admission, HCQ, CSA, and MMF.

### 3.2 Circulating lymphocyte subset profile, by CMV infection status

The total lymphocytes and lymphocyte subsets including CD4+ T cells, CD8+ T cells, CD19+ B cells, and CD56+CD16+ NK cells, were compared between CMV-infected patients and those in the control group (Table 2). We also showed the total lymphocytes and lymphocyte subset counts of sex- and age-matched healthy individuals as a reference in Table 2. The levels of total lymphocytes and different lymphocyte subsets were similar between patients in the uninfected control group and the CMV viremia group. However, by comparison, patients in the CMV disease group had significantly lower levels of total lymphocytes (680 ± 513 cells/μL vs 1520 ± 101 cells/μL, *P* < .001), CD4+ T cells (189 ± 192 cells/μL vs 571 ± 447 cells/μL, *P* < .001), CD8+ T cells (300 ± 271 cells/μL vs 583 ± 434 cells/μL, *P* < .001), CD19+ B cells (130 ± 174 cells/μL vs 254 ± 237 cells/μL, *P* = .015), and CD56+CD16+ NK cells (33 ± 36 cells/μL vs 55 ± 38 cells/μL, *P* = .028) compared with patients in the uninfected control group.

**Table 2:**
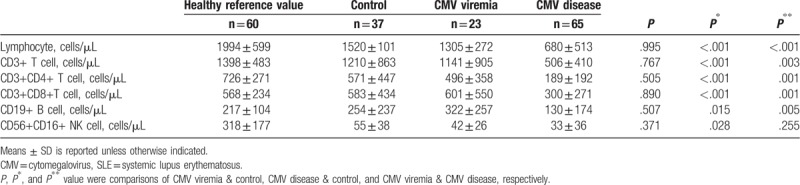
The variation of lymphocyte subsets among different SLE groups.

### 3.3 Association between circulating lymphocyte subsets and CMV infection status

Our univariate logistic regression model (Table 3) demonstrated an inverse association between CMV disease and total lymphocyte count (odds ratio [OR] 0.998, 95% confidence interval [CI] 0.997–0.999, *P* < .001), CD4+ T cells (OR 0.994, 95% CI 0.992–0.997, *P* < .001), CD8+ T cells (OR 0.997, 95% CI 0.995–0.999, *P* = .001), CD19+ B cells (OR 0.998, 95% CI 0.996–1.000, *P* = .033), and CD56+CD16+ NK cells (OR 0.991, 95% CI 0.982–1.000, *P* = .052). In the multivariable regression analysis, the total lymphocyte count (OR 0.999, 95% CI 0.998–1.000, *P* = .007) and CD4+ T cell count (OR 0.995, 95% CI 0.992–0.998, *P* = .003) remained negatively associated with CMV disease. However, no significant associations were observed between CMV viremia and lymphocyte subsets in both the univariate and multivariable logistic regression models.

**Table 3:**
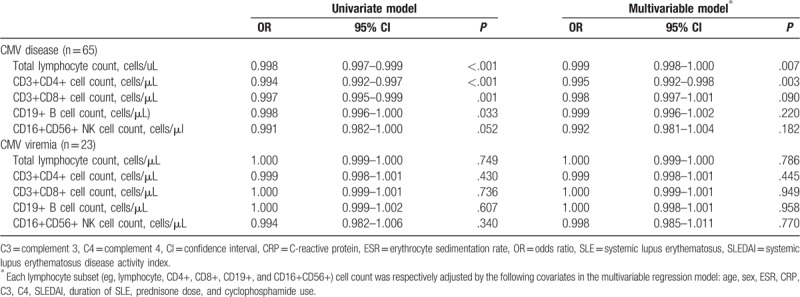
Association between lymphocyte subsets and CMV infection in logistic regression analyses.

## 4 Discussion

This pilot study uniquely explores the differential relationship between lymphocyte subsets and the presence of CMV disease versus CMV viremia among patients with SLE, compared with uninfected patients with SLE. Building upon our previous work demonstrating that lymphocyte counts and lymphocyte subsets are significantly decreased among patients with SLE who present with OIs,^[17]^ our current findings demonstrate that total lymphocyte and CD4+ T-lymphocyte counts are significantly decreased among SLE patients with CMV disease, but not in those with CMV viremia alone or in patients without infection, suggesting additional studies are worthwhile to explore peripheral CD4+ T-cell lymphopenia as a potential biomarker for distinguishing the 2 clinical states in this population.

CMV infection can cause both systemic and organ-specific disease, not only through the direct cytopathic effects of viral replication in host cells, but also through inflammatory processes trigger SLE flare.^[26]^ Furthermore, the clinical features of CMV infection themselves sometimes mimic SLE flare. Finally, the use of corticosteroids and/or immunosuppressants can also inhibit immunologic function and reduce inflammatory and febrile responses,^[9,27]^ leading a proportion of symptomatic patients with CMV infection to be asymptomatic. Therefore, based upon clinical features alone, it may still be challenging for clinicians to recognize active CMV infection and distinguish it from SLE flare. Previous studies of risk factors for CMV infection and associated treatment strategies have mainly focused on transplant^[28]^ and acquired immune deficiency syndrome (AIDS) patients.^[29]^ Among patients with AIDS and a history of CMV, secondary prophylaxis of CMV infection has been established as a beneficial strategy to avoid the severe consequences of CMV infection while the CD4+T cell count remains is below 100/μL. However, to the best of our knowledge, very few data have examined the potential application of lymphocyte subsets such as CD4+ T cell count as an immunological biomarkers for the management of CMV infection in patients with SLE.

To date, there is still limited knowledge regarding risk factors for incident CMV disease among patients with rheumatologic disease. Takizawa and colleagues carried out a 5-year, large-scale multicenter retrospective survey^[30]^ among 7377 patients with rheumatologic illness. Their study showed that the highest incidence of CMV infection was observed in patients with SLE (n = 151), and 3 quarters of CMV-infected patients presented with CMV disease. Compared to patients with CMV viremia, most patients with CMV disease had received more immunosuppressive therapy in the previous year, in particular pulse steroids, high dose steroids or CPM. In that study, risk factors strongly associated with the development of CMV infection included lymphopenia, older age (>59 years), and the use of pulse steroids. Another retrospective study in Taiwan^[31]^ found that compared with patients without CMV infection, those with CMV infection had a higher mean prednisolone dose (25.9 vs 9.0 mg/d, *P* = .006), higher rates of AZA use (35% vs 5.6%, *P* = .045), and lower lymphocyte counts (743 vs 1062 cells/μL, *P* = .175). A study among pediatric patients with SLE^[32]^ also showed the proportion of patients with lymphocyte counts <500 cells/μL (70% vs 51%, *P* < .001) and prednisone use (100% vs 43%, *P* = .003) were significantly greater among SLE patients with CMV infection as compared with noninfected patients. In our study, 65 (73.8%) CMV-infected patients had CMV disease. Compared with noninfected patients, the frequency of usage and dose of CPM were higher among SLE patients presenting with CMV disease, but not significantly different from SLE patients with CMV viremia. We would not assess the impact of AZA on CMV infection due to insufficient numbers. The total lymphocyte counts were lower (680 vs 1520 cells/μL, *P* < .001) in CMV disease, but remained stable in CMV viremia, as compared with those without infection.

Risk factors for CMV disease among patients with SLE, such as prednisone usage, immunosuppressant usage, age, and disease duration, vary among different studies due to differences in study design and sample size. However, lymphopenia is consistently observed, as is supported by our findings. Further analysis of lymphocyte subsets (CD4+ T cells, CD8+ T cells, CD19+ B cells, and CD56+CD16+ NK cells) using flow cytometry revealed variations in levels of lymphocyte subsets in setting of CMV infection status, ranging from viremia to organ related disease. Compared with patients without infection, B cells, T cells, and NK cells in SLE patients with CMV disease were notably decreased. After adjusting for relevant covariates, logistic regression analysis demonstrated a negative association remained between CD4+ T cell levels and CMV diseases status. By contrast, the downregulation of lymphocyte subsets among patients with CMV viremia in our study was very mild.

A study^[33]^ from India showed that the CD4+ T cell percentage in pretherapy SLE patients is higher than the CD8+ T cell percentage. In vitro stimulation of peripheral blood mononuclear cells from pretherapy SLE patients with CMV-specific antigen led to a significant further increase in CD4 T cell percentage, dominated by CD4+ memory T cells. This increase in CD4+ memory cells and their related cytokines could play a role in SLE disease progression. However, the study also observed that after 6 months of immunosuppressive therapy, the CD4+T cell percentage was decreased in peripheral blood mononuclear cells from SLE patients that did not receive CMV antigen stimulation, and CD4+ memory T cells were still augmented. The findings above indicate that immunosuppressive therapy may diminish the autoimmune response, but the pool of memory T cells and the overproduction of cytokines still exist and may play an important role in activating CMV-specific responses and organ damage in SLE patients. Therefore, early identification of, and targeted therapy for CMV infection remains an important part of the treatment strategy for this vulnerable population, especially after immunosuppressive treatment. This may partially explain the association between decreased CD4+ T cell and CMV disease among SLE patients in our study.

It is well-known that CD4+ T cells play a central role in coordinating innate and adaptive immune responses, as demonstrated by the susceptibility to pathogenic and OIs resulting from primary or acquired CD4+ T cell immunodeficiency. In cases such as immunosuppressive therapy or HIV infection, decreases in the CD4+ T cell count can directly contribute to the development of OIs.^[34]^ CD4+ T cells play a central role in modulating host immune responses to pathogens, and along with CD8+ T cells, make up the majority of T lymphocytes.^[35]^ CD4+ T cells carry out multiple functions, including the activation of B-lymphocytes and cytotoxic T cells.^[36]^ These “helper” CD4+ T cells do not neutralize infections but rather trigger the body's response to infections. In response, cytotoxic CD8+ T cells produce substances that help fight against viruses and other foreign invaders. CD4+ T cells, after being activated and differentiated into distinct effector subtypes (ie, Th1 cells, Th2 cells, and Th17 cells),^[36]^ play a central role in activating macrophage and dendritic cells during initial infections with parasites, bacteria, or viruses. It is possible that significant alteration in CD4+ T cell counts due to underlying disease activity, SLE-related treatments, or genetic factors, results in downstream alterations in other lymphocyte subset counts as well.

Given the complexity involved in managing patients with SLE, particularly those hospitalized with acute illness, identifying patients who are at greatest risk for CMV disease and determining optional therapeutic strategies remain critically important. Our findings suggest that the lymphopenia, especially decreased CD4+ T cell counts, may be associated with presence of CMV disease among patients with SLE. Further prospective multicenter cohort studies are needed to establish the potential role of the lymphocyte and/or CD4+ T cell as a biomarker for the development of CMV disease among SLE patients.

Our study has a number of limitations. First, our study is cross-sectional and, therefore, we cannot infer a causal relationship between the level of different lymphocyte subsets, especially CD4+ T cell count, and occurrence of CMV disease. Second, given this study was carried out at a single center among hospitalized patients, our findings cannot necessarily be generalized to outpatients with SLE or those from other regions. Third, results of CMV-IgM and IgG tests were not analyzed in this study because they were not measured systematically for all patients as part of the diagnostic workup for CMV infection. In the complex immune setting of SLE, CMV-IgM, or IgG testing may result in false-negative and false-positive results, therefore diagnoses of CMV infection were made based upon CMV DNA viral loads. Finally, as this was a pilot study, the number of patients with SLE enrolled in this study was limited and therefore these findings should be confirmed in a larger prospective study.

## Acknowledgments

We are grateful to all the SLE patients who participated in this study. We also acknowledge the staff at the Rheumatology Department of Peking Union Medical College Hospital. Finally, we thank Dr. Insoo Kang (Yale School of Medicine, CT, USA) for his input during the preparation of this manuscript.

## Author contributions

**Conceptualization:** Taisheng Li.

**Data curation:** Ling Qin, Zhifeng Qiu, Taoran Geng, Jing Xie.

**Investigation:** Taoran Geng, Lu Wan, Taisheng Li.

**Methodology:** Ling Qin, Evelyn Hsieh, Taisheng Li.

**Project administration:** Jiuliang Zhao, Taisheng Li.

**Resources:** Jiuliang Zhao, Xiaofeng Zeng.

**Supervision:** Taisheng Li.

**Visualization:** Xiaofeng Zeng, Jean Pierre Routy, Taisheng Li.

**Writing – original draft:** Ling Qin.

**Writing – review and editing:** Ling Qin, Evelyn Hsieh, Xiaofeng Zeng, Rayoun Ramendra, Jean Pierre Routy, Taisheng Li.
